# A Computer-Based Screening Method for Distress in Patients With Multiple Sclerosis: A Feasibility Study

**DOI:** 10.2196/resprot.3098

**Published:** 2014-06-04

**Authors:** Rosa E Boeschoten, Bernard MJ Uitdehaag, Patricia van Oppen, Vincent de Groot, Emma H Collette, Kim AA Bakker, Chris H Polman, Joost Dekker

**Affiliations:** ^1^Department of PsychiatryVU University Medical Center and GGZinGeestAmsterdamNetherlands; ^2^EMGO Institute for Health and Care ResearchVU University and VU University Medical CenterAmsterdamNetherlands; ^3^Department of NeurologyVU University Medical CenterAmsterdamNetherlands; ^4^Department of Epidemiology and BiostatisticsVU University Medical CenterAmsterdamNetherlands; ^5^Department of Rehabilitation MedicineVU University Medical CenterAmsterdamNetherlands; ^6^Department of Medical PsychologyVU University Medical CenterAmsterdamNetherlands

**Keywords:** multiple sclerosis, distress, computer-based, screening, feasibility, depression, anxiety, cognitive functioning

## Abstract

**Background:**

In multiple sclerosis (MS) patients, symptoms of anxiety, depression, pain, and cognitive impairment are highly prevalent and contribute to lower wellbeing. As these physical and psychological symptoms of distress often stay unnoticed, regular screening could offer possibilities to identify and refer impaired patients to appropriate care.

**Objective:**

The aim of our study was to pilot a new computer-based method in 43 MS patients to efficiently screen for a variety of psychological and physical symptoms of distress.

**Methods:**

Data on feasibility and psychological and physical distress (anxiety, depression, fatigue, physical disability, cognitive functioning) were collected via a touch screen computer. Referral to psychosocial care and rehabilitation was retrospectively checked.

**Results:**

The results demonstrated that most patients (35/40, 88%) considered the screening meaningful and the system easily usable (37/40, 93%). Average completion time of the screening was below 8 minutes. Many patients (35/40, 88%) had elevated distress levels, of whom the majority was referred.

**Conclusions:**

These findings imply that computer-based screening for MS-related distress incorporated in clinical care is feasible and aids to identify psychological or physical needs. A randomized controlled trial with follow-up should address whether this screening method could be more effective than routine care, and whether it can improve costs and efficiency of care.

## Introduction

Multiple sclerosis (MS) is a chronic inflammatory disease of the central nervous system that can have a great impact on a patient’s life. In multiple sclerosis patients, symptoms such as depression, anxiety, fatigue, pain, and cognitive dysfunction, are highly prevalent both in early and more advanced disease stages and related to lower quality of life [1-3]. In the present study, we focus on this wide variety of physical and psychological symptoms impairing MS patients in their daily activities, and refer to them with the umbrella term “distress”. Although treatments are available that can help to minimize some of these symptoms, still much distress remains unrecognized and untreated [2,4]. Consequently, it has been recommended that, with each visit to the neurologist or clinic, neurological nurses should screen and evaluate the level of distress in MS patients [5].

In clinical care, routine screening techniques can help to enable adequate recognition of distress and referral to appropriate care. Lately, successful initiatives of computer-assisted data collection in health care have increased. Advantages are high compliance rates, rapid completion and processing, and immediately available results [6-11]. The aim of the present study was to pilot a computer-based screening method, which can be easily incorporated into clinical care to support MS nurses in identifying psychological or physical needs of MS patients.

## Methods

### Patients and Procedure

From February to August 2012, consecutive MS patients who visited the MS nurse of the Department of Neurology of the VU University Medical Center in Amsterdam, the Netherlands, were asked to complete a computer-based screening with six self-report questionnaires. Patients were mainly referred to the MS nurse by their neurologist after their first visit, a standard procedure for patients who remain under our care, or could make a request for consultation themselves. Consultation with the MS nurse was aimed at getting acquainted, providing information on MS and treatment, and discussing further assistance if required.

**Figure 1:**
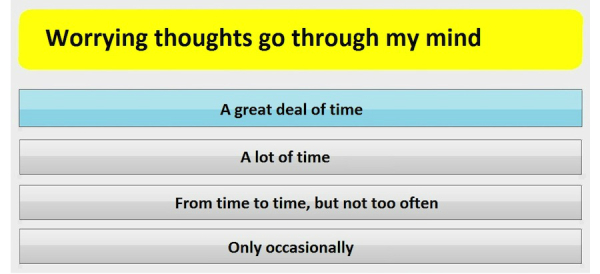
Screenshot of one of the questions of the Hospital Anxiety and Depression Scale as presented to the patient on the touchscreen (translated from Dutch to English).

One week before the visit, patients were invited by telephone and letter to participate in the pilot. Fifteen minutes before the consultation, nursing staff assisted the patient to the touch screen computer in a private room to fill out the questionnaires. The patient identification number was filled in, which was linked with the hospital database that contains general data on the patient’s age, gender, and disease history. Then, questions on psychological and physical distress followed by questions on satisfaction about the screening procedure were presented to the patient on the computer screen one by one (Figure 1). The patient answered by touching the appropriate response on the screen and then moved on to the next question. More details of the software and computer system have been described elsewhere [9].

**Figure 2:**
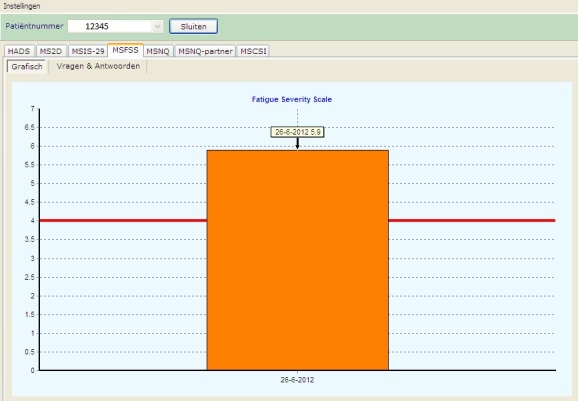
Screenshot of the patient score on one of the questionnaires (Fatigue Severity Scale) as presented to the nurse. The red line indicates the cut-off value.

When patients finished the screening, they were assisted to visit the MS nurse in another room. By using the patient identification number, the nurse had direct access to the results that were displayed in graphs on her computer screen (Figure 2). At the end of the pilot project, the MS nurse was asked to evaluate the screening.

### Measures

#### Feasibility

Compliance rate and time needed to complete the questions were recorded. Patient satisfaction regarding the system and procedure was evaluated by seven self-designed questions and a 10-point visual analogue scale (VAS). Also the MS nurse was presented with comparable questions of satisfaction on paper.

#### Distress

We used questionnaires that have been shown to be reliable, valid, and frequently used in clinical practice and/or research in MS. Clinical relevant cut-offs based on literature or clinical practice were used for all questionnaires. Anxiety and depression were measured with the Hospital Anxiety and Depression Scale (HADS, each subscale cut-off>7) [12], fatigue with the Fatigue Severity Scale (FSS, cut-off≥4) [13], and cognitive functioning with the Multiple Sclerosis Neurological Questionnaire (MSNQ, cut-off>27) [14]. The Multiple Sclerosis Impact Scale-29 (MSIS-29) was used to explore the impact of MS on physical (cut-off>60) and psychological wellbeing (cut-off>24) [15]. Finally, patients were asked to fill in the VAS health thermometer from the EuroQol-5D (EQ-5D). The EQ VAS self-rating records the respondent’s own assessment of their health status on a vertical VAS where the endpoints are labeled “best imaginable health state” (100) and “worst imaginable health state (0)” [16].

#### Referral

Several weeks after the consultation, we explored patients’ medical files. Referrals were retrospectively coded to social workers, psychologists, physiatrists, physiotherapists, and occupational therapists.

## Results

### Feasibility

Of the 43 referred patients, 2 patients did not give consent for scientific documentation and 1 was excluded because he was physically unable to complete the screening questionnaires. It took the 40 remaining patients on average 7.4 minutes (median=6.8; interquartile range=3.1) to complete the 66 questions on distress. This was evaluated as “little time” by 36 of 40 patients (90%) and the majority (37/40, 93%) reported that the equipment was easy to use and experienced the screening as meaningful (35/40, 88%). On average, the screening was graded 7.5 (range 3-10, N=38).

The MS nurse evaluated the screening positive on the VAS (7.5). She was satisfied with the quality and content, the system was easy to use and it took her little time to consult the screening data. The screening facilitated her work and helped her to more specifically focus on actual problems to be addressed, including unmentioned problems that could be overlooked easily.

### Outcome Measures

**Figure 3:**
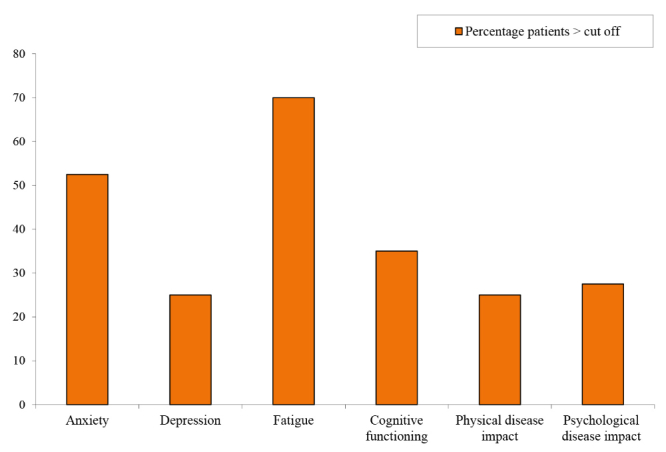
Percentage of MS patients (N=40) with high level of psychological or physical distress.

For the total group (N=40), the mean HADS-score for anxiety was 8.3 (SD 3.6) and depression 5.4 (SD 3.8). Mean FSS and total MSIS-29 scores were 5.0 (SD 1.6) and 67.7 (SD 25.3), respectively. Mean MSNQ-score was 23.4 (SD 12.2). On average, patients gave their general health 66 (SD 17.7) points out of 100. A large part of patients (35/40, 88%) had scores above cut-off, indicating high levels of distress*.* More specifically, Figure 3 shows that 21 of 40 patients (53%) met criteria for anxiety. A remarkably lower percentage of patients met the criteria for depression (10/40, 25%). Fourteen of 40 patients (35%) had significant cognitive complaints, 10 of 40 patients (25%) experienced a high physical impact of MS, and 28 of 40 patients (70%) met criteria for significant fatigue.

### Referral

Some patients reported already suitable treatment for their distress. However, Table 1 shows that the majority was referred by the MS nurse to psychosocial care or rehabilitation.

**Table 1:** Percentage referred and treated MS patients with low and high levels of distress (N=40).

		HADS^a^		MSIS-29^b^		FSS^c^		MSNQ^d^	
		<cut off (n=18), %	>cut off (n=22), %	<cut off (n=25), %	>cut off (n=15), %	<cut off (n=12), %	>cut off (n=28), %	<cut off (n=26), %	>cut off (n=14), %
No referral		28	18	32	7	34	18	31	7
Suitable care		17	14	12	20	8	14	19	0
**Referral**		55	68	56	73	58	68	50	93
	Referral only	17	18	16	20	8	25	15	29
	Treatment after referral	38	50	40	53	50	43	35	64

^a^Hospital Anxiety and Depression Scale, measures anxiety and depression

^b^Multiple Sclerosis Impact Scale-29, measures Physical and Psychological disease impact

^c^Fatigue Severity Scale, measures fatigue

^d^Multiple Sclerosis Neurological Questionnaire; measures cognitive functioning

## Discussion

### Principal Findings

This pilot study shows that computer-based screening is a feasible way to detect psychological and physical distress in MS patients in clinical care, and could support MS nurses in their work. It constitutes an easy way to administer questionnaires and processing data that can be directly available to patients and nurses. The computer-based method we demonstrated here can be easily adapted for routine screening. It would be suitable for application on personal mobile devices or via an Internet website, offering patients the possibility to complete it in their own time and pace, improving costs and efficiency of care.

Regular screening offers possibilities to identify and refer impaired patients to appropriate care as early as possible and monitor distress. Also, screening could increase patient awareness that their experienced distress can be related to MS, which might decrease barriers to request appropriate treatment. Next to clinical use, data collection could be suitable for scientific documentation.

### Conclusions

MS patients appear to be willing to complete a computer-based screening. Average completion time of our assessment was comparable with similar initiatives (5-8.7 minutes) [6-8]. Many patients showed elevated levels of distress, and were referred to further care. However, the number of referred patients with minimal distress was disproportionally high. Moreover, the results do not provide us with a complete overview of prescribed medication and referrals other than psychosocial and revalidation care. In addition, whether relevant needs of MS patients are covered by this procedure is still unclear because our study concerns a pilot design using an uncontrolled unselected sample. Therefore, the findings of this study should be used with caution. A randomized controlled trial with longer follow-up should reveal whether routine screening, in comparison to routine care, is effective in detecting distress that would otherwise remain unnoticed, and results in appropriate referrals, adequate treatments, and improved distress outcomes.

## Abbreviations

EQ: EuroQol
FSS: fatigue severity scale
HADS: hospital anxiety and depression scale
MS: multiple sclerosis
MSIS-29: multiple sclerosis impact scale-29
MSNQ: multiple sclerosis neurological questionnaire
VAS: visual analogue scale
